# Videogame-based group therapy to improve self-awareness and social skills after traumatic brain injury

**DOI:** 10.1186/s12984-015-0029-1

**Published:** 2015-04-11

**Authors:** Roberto Llorens, Enrique Noé, Joan Ferri, Mariano Alcañiz

**Affiliations:** Instituto Interuniversitario de Investigación en Bioingeniería y Tecnología Orientada al Ser Humano, Universitat Politècnica de València, Camino de Vera s/n, 46022 Valencia, Spain; Servicio de Neurorrehabilitación y Daño Cerebral de los Hospitales NISA, Fundación Hospitales NISA, Río Tajo 1, 46011 Valencia, Spain; Ciber, Fisiopatología Obesidad y Nutrición, CB06/03 Instituto de Salud Carlos III, Univesity of Jaume I, Av. Sos Baynat s/n, 12071 Castellón, Spain

**Keywords:** Self-awareness, Social skills, Traumatic brain injury, Rehabilitation, Neuropsychology, Virtual reality

## Abstract

**Background:**

This study determines the feasibility of different approaches to integrative videogame-based group therapy for improving self-awareness, social skills, and behaviors among traumatic brain injury (TBI) victims and retrieves participant feedback.

**Methods:**

Forty-two adult TBI survivors were included in a longitudinal study with a pre- and post-assessments. The experimental intervention involved weekly one-hour sessions conducted over six months. Participants were assessed using the Self-Awareness Deficits Interview (SADI), Patient Competency Rating Scale (PCRS), the Social Skills Scale (SSS), the Frontal Systems Behavior Scale (FrSBe), the System Usability Scale (SUS). Pearson's chi-squared test (*χ*^2^) was applied to determine the percentage of participants who had changed their clinical classification in these tests. Feedback of the intervention was collected through the Intrinsic Motivation Inventory (IMI).

**Results:**

SADI results showed an improvement in participant perceptions of deficits (*χ*^2^ = 5.25, p < 0.05), of their implications (*χ*^2^ = 4.71, p < 0.05), and of long-term planning (*χ*^2^ = 7.86, p < 0.01). PCRS results confirm these findings (*χ*^2^ = 5.79, p < 0.05). SSS results were also positive with respect to social skills outcomes (*χ*^2^ = 17.52, p < 0.01), and FrSBe results showed behavioral improvements (*χ*^2^ = 34.12, p < 0.01). Participants deemed the system accessible (80.43 ± 8.01 out of 100) and regarded the intervention as interesting and useful (5.74 ± 0.69 out of 7).

**Conclusions:**

Integrative videogame-based group therapy can improve self-awareness, social skills, and behaviors among individuals with chronic TBI, and the approach is considered effective and motivating.

## Background

Self-awareness (SA) impairment refers to a reduced ability to appraise one’s strengths and weaknesses and the consequent implications of this tendency on present and future life activities [1,2]. SA is a broad and complex concept that implies the interaction of various cognitive processes and primary human psychological functions [3]. Pathogenesis processes underlying this symptom are still unknown. Although it has been linked to frontal lobe dysfunction [4,5], recent studies suggest that SA impairment may result from a breakdown of functional interactions between nodes within the fronto-parietal control network [6]. Such a diffuse neuroanatomical distribution renders SA extraordinarily vulnerable to deleterious effects of diffuse axonal injury arising after the occurrence of traumatic brain injury (TBI). SA impairment has been reported as a common symptom following the occurrence of TBI, manifesting in 45% to 97% of all cases [1].

In the neuropsychological domain, TBI survivors can exhibit attention span, memory, and reasoning impairments that in turn can hinder their self-monitoring skills [7]. All of these deficits together with possible concurrent problems of emotional coping and acceptance can result in SA impairment. SA deficits can have serious effects on everyday functioning. Patients may not understand the purpose of their participation in a neurorehabilitation program, and in turn may be unmotivated, uncooperative, and irritable [8]. SA impairment can also lead patients to set unrealistic goals [9], thus hindering their functional competence [10] and process of vocational reentry [1]. In addition, emotional SA impairment can affect one’s awareness of differences between oneself and others, adaptation to other perspectives, and means of modulating another person’s behaviors [11]. Consequently, SA deficits can have a significant impact on social skills and emotional regulation [12,13], rendering community integration difficult [14].

A three-layer pyramidal model is typically used to conceptualize this phenomenon [15]. The model differentiates between: 1) intellectual awareness, or the ability to perceive a skill as impaired and recognizing their implications; 2) emergent awareness, or the ability to realize ongoing complications; and 3) anticipatory awareness, or the ability to anticipate future problems derived from the impairment. This model has recently been extended to consider metacognition [16]. Various strategies based on this model have been proposed to mitigate effects of SA impairment [17], and several recommendations have been presented from available evidence [18]. Educational formats have been shown to improve intellectual awareness by increasing knowledge of residual impairments and their implications [17]. Interestingly, group therapy programs, including game-based programs [19,20], have been reported to reinforce the benefits of this form of intervention [21,22]. SA interventions often involve task performance feedback [23], either verbally through the participation of a therapist or visually through the use of videotaped sessions [24-26], to improve intellectual, emergent, and anticipatory awareness. Other programs have explored the effectiveness of behavioral interventions, confrontational techniques, motivational interviews, counseling, and psychotherapy [27]. However, while there is increasing evidence that SA interventions following TBI are effective, further research is needed to confirm reported results. Moreover, limited research has been conducted on interactions between SA and social skills following the occurrence of a TBI.

Basing on existing evidence [17] and recommendations [18], we present a videogame-based group therapy program that focuses on social interaction to SA improvement. This experimental intervention focuses on the use of social and metacognitive skills for reasoning, problem-solving, and planning, and incorporated feedback from peers and therapists. The objectives of this study are threefold: first, to determine the feasibility of this program for TBI individuals; second, to quantify how the approach affects social skills and behaviors associated with frontal lobe damage; and to retrieve participant feedback on the intervention.

## Methods

### Setting

The study was conducted at the Servicio de Neurorrehabilitación y Daño Cerebral of NISA Hospital Valencia al Mar in Spain. Ethical approval for the study was granted by the medical center’s Institutional Review Board.

### Participants

Outpatient neurorehabilitation program participants were considered as potential study participants. The following inclusion criteria were used: 1) moderate to severe TBI according to the TBI Mayo Classification System [28]; 2) the emergence from posttraumatic amnesia over no less than three months; 3) aged ≥ 25 and ≤ 65 years; 4) chronicity > six months; 5) absence of cognitive impairment as specified by the Mini-Mental State Examination [29] > 23; and 6) fairly strong language comprehension, as specified by the Mississippi Aphasia Screening Test [30] > 45. The following exclusion criteria were used: 1) patients with behavioral problems that prevent their participation in group therapy; 2) patients with visual or hearing impairments that limit their degree of interaction; 3) patients with severe dementia. All participants who agreed to take part in the study were required to provide informed consent.

### Measures

The Self-Awareness Deficits Interview (SADI) approach was used to measure intellectual awareness and metacognition variables [9]. The SADI is a three-component interview that assesses one’s awareness of deficits and their functional implications and one’s ability to set realistic goals. Each component is scored by a therapist on a four-point Likert scale ranging from zero (no awareness) to three (complete awareness). Participants were classified as exhibiting altered or normal SA following recommendations of previous studies [21]. More specifically, lower scores (0–1) reflect moderate to high SA. Conversely, higher scores (2–3) reflect a tendency to deny or minimize the extent of difficulties experienced.

As recommended by the literature, a second measure of SA was added. The Patient Competency Rating Scale (PCRS) was used not only as a secondary measure of intellectual awareness but also to assess social skills [31]. The PCRS compares patient self-ratings of competencies in 30 areas related to activities of daily living, cognitive functioning, social skills, and emotional regulation with those provided by a relative or therapist. Each item is rated on a five-point Likert scale ranging from one (impossible to do) to five (easy to do). In this study, a relative or caregiver was responsible for completing the scale. Three scores were obtained from the scale: the number of items wherein the subject's rating was higher than the respondent's rating; the number of items wherein the respondent's rating was higher than the subject’s rating; and the number of items wherein ratings were identical. Subjects were then classified as presenting not altered SA if the number of identical responses was higher than number of the differing responses and as exhibiting altered SA otherwise.

The Social Skills Scale (SSS) was used to assess social skills [32]. The scale explores how social skills regulate individual behaviors in specific situations. The SSS is a self-administered 33-item questionnaire divided into six components: self-expression in social situations, defense of one’s own rights, expression of anger or disagreement, rejection and interaction cutoff, request making, and initiation of positive interactions with persons of the opposite sex. Each item is scored on a four-point Likert scale ranging from one (not representative at all) to four (totally representative). Raw scores were converted to percentile scores according to the scale manual. Percentile scores greater than 16 were considered not altered. Scores ranging from two to 16 were considered altered. Scores fewer than two were considered very altered.

The Frontal Systems Behavior Scale (FrSBe) was used to measure three frontal system behavioral syndromes: apathy, disinhibition, and executive dysfunction [33]. The FrSBe is a 46-item questionnaire that is administered to a patient and relative to evaluate the patient’s condition before the injury and before and after the treatment. The disparity between observer and patient scores serves as a metacognitive measure of SA [33]. Items are rated on a five-point Likert scale ranging from one (almost never) to five (almost always). All scores were converted to T-scores corrected for age, education, and gender. T-scores of less than 60 were considered not altered, T-scores ranging from 60 to 64 were considered of borderline significance, and T-scores of 65 or above were considered clinically significant.

In addition to the clinical scales, the System Usability Scale (SUS) [34] and Intrinsic Motivation Inventory (IMI) [35] were administered to all participants following the treatment. The SUS is a simple ten-item scale that serves as a global assessment of subjective usability. The SUS employs a Likert scale with scores ranging from 0 to 100. The IMI is a multidimensional questionnaire structured into various subscales. Each subscale includes different questions rated on a seven-point Likert scale. In this study, the IMI was used to assess participant interest/enjoyment, perceived competence, pressure/tension, and value/usefulness measures. Scores approaching seven in each subscale represent positive values in terms of motivation, with the exception of the pressure/tension subscale, for which high scores represent high levels of tension.

### Procedure

**Figure 1:**
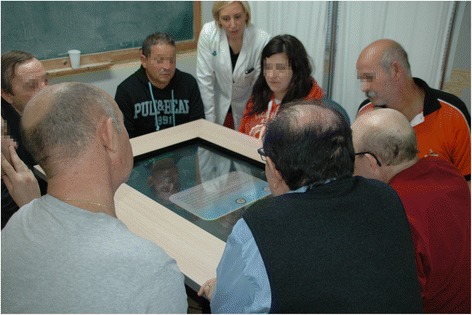
**Participants in the middle of a session.** Participants interacting with the videogame. The team in the top of the picture has the turn. A participant belonging to the team located in the opposite side of the multitouch table is reading a question card about reasoning (blue card): “Rose has just left the ICU. Her relatives are a little worried because she is being hospitalized in a neurorehabilitation unit next week. What would you tell them to calm them down (functioning of the unit, admission to the floors, rehabilitation process, etc.)?” Participants who have the turn have to listen to the question, answer it, and justify the answer. After that, the therapist will involve the other participants in a debate about the question.

Participants were divided into groups of eight people that present similar cognitive conditions, according to therapists’ perception. In each group, participants were divided into teams of two. Hence, each group consisted of four pairs of individuals sitting on different sides of a table playing a digital board game under the supervision of an experimented neuropsychologist. A multi-touch screen embedded into a conventional table displayed the game board, and participants engaged by touching elements presented on the screen (Figure 1).

The objective of the videogame was to reach the top of a mountain. Each team strove to reach the summit first by correctly answering questions presented on cards as fast as possible, as in a conventional board game. Question cards included four different types of questions:

- Knowledge (red cards): Anatomical and pathological matters. For instance: ‘Lesions of the right hemisphere often cause language problems. True or false?’
- Reasoning (blue cards): Situational exercises. For instance: ‘Your friends are talking at the same time and you cannot follow the conversation. What do you do?’
- Action (green cards): Role-playing exercises. For instance: ‘Sometimes, a brain injury can cause motor problems in one side of the body. Put your socks and shoes on using only one hand’
- Cohesion (yellow cards): Jokes and sayings. For instance: ‘Solve this riddle’.

On their turn, each team rolled a dice to move their game piece to the corresponding game board square and then answered a random question from those categorized under the square color. Teams answering correctly moved on to the following square (without answering a question). Some squares distributed throughout the game board caused teams lose a turn (avalanche, bear attack, etc.). Other squares granted participants special abilities to steal another participant’s question or to assign their own question to another participant.

To promote participant engagement, the card text was displayed to the opposite side of the table. In turn, the group on the opposite side of the table read the other team’s question aloud for the other group to answer. After an answer was given, the neuropsychologist involved all game participants and alternative answers were discussed. The therapist encouraged participants to play roles and confront their limitations and gave verbal feedback and support with each turn.

All of the participants engaged a one-hour session each week over six months. Two experimented therapists conducted the intervention sessions. All of the sessions of each group were conducted by the same neuropsychologist. A different experimented therapist assessed the condition of the participants before (baseline) and after the intervention. Assessments were conducted in the week before the intervention and in the week after its completion.

### Statistical analysis

Pearson's chi-squared test (*χ*^2^) was applied to determine the percentage of participants who had changed their clinical classification. The α level (two-sided) was set at 0.05 for all of the analyses. All analyses were computed using SPSS for Mac, version 15 (SPSS Inc., Chicago, USA).

## Results

A total of 73 TBI survivors were enrolled in the neurorehabilitation program for the duration of the study. Of these, 21 subjects did not satisfy the study inclusion criteria, and four declined to participate in the study. The remaining 48 participants (six groups) were included in the study. Six participants dropped out of the study after being discharged from the neurorehabilitation program (n = 5) or due to medical complications (n = 1). These individuals were replaced with other participants to maintain the two-person team format, though their results are not included in the study. The results for the remaining 42 participants are presented in Table 1.

**Table 1:** **Characteristics of the participants**

**Characteristic**	**Values**
*Gender (n, %)*	
Males	27 (64.3%)
Females	15 (35.7%)
*Age (years)*	41.71 ± 13.49
*Chronicity (days)*	227.95 ± 50.20
*Cause of the injury (n, %)*	
Traffic accident	33 (78.6%)
Workplace accident	5 (11.9%)
Fall	4 (9.5%)
*Glasgow comma scale (n, %)*	
Moderate (9–12)	3 (7.1%)
Severe (<9)	39 (92.9%)

Age and chronicity are defined in terms of mean and standard deviation. Gender, cause of the injury, and Glasgow Comma Scale are expressed as number of participants and percentage of the total number of participants.

The results show that the participants benefited from the intervention across all clinical measures (Table 2). No negative tendencies were detected in any scale. With regards to SA, the SADI showed improvements in perceptions of deficits (*χ*^2^ = 5.25, p < 0.05) and their implications (*χ*^2^ = 4.71, p < 0.05) and improvements in planning skills (*χ*^2^ = 7.86, p < 0.01). More specifically, at the beginning of the intervention, 16 participants presented limitations in perceiving their deficits, 24 participants presented difficulties in perceiving their disabilities, and 31 participants presented difficulties in setting realistic goals. After the treatment, almost all of the participants (n = 39) perceived their deficits properly, 12 participants still showed limitations in perceiving their disabilities, and 19 participants still struggled with establishing realistic goals. The PCRS results confirm these improvements (*χ*^2^ = 5.79, p < 0.05). According to this scale, one third of the sample (n = 14), altered at the baseline, was classified as not altered after the intervention.

**Table 2:** **Clinical data**

**Scale**	**Initial assessment**			**Final assessment**			**Significance**
*SADI (n, %)*	Altered		Not altered	Altered		Not altered	
Perception of deficits	16 (38.1%)		26 (61.9%)	3 (7.1%)		39 (92.9%)	*χ* ^2^ = 5.25, p = 0.022
Perception of disability	24 (57.1%)		18 (42.9%)	12 (28.6%)		30 (71.4%)	*χ* ^2^ = 4.71, p = 0.030
Realistic plan-making	31 (73.8%)		11 (26.2%)	19 (45.2%)		23 (54.8%)	*χ* ^2^ = 7.86, p = 0.005
*PCRS (n, %)*	Altered		Not altered	Altered		Not altered	*χ* ^2^ = 5.79, p = 0.016
	32 (76.2%)		10 (23.8%)	18 (42.9%)		24 (57.1%)	
*SSS (n, %)*	Very altered	Altered	Not altered	Very altered	Altered	Not altered	*χ* ^2^ = 17.52, p = 0.000
	6 (14.3%)	25 (59.5%)	11 (26.2%)	0 (0%)	14 (33.3%)	28 (66.7%)	
*FrSBe (n, %)*	Clinically significant	Borderline impairment	Not altered	Clinically significant	Borderline impairment	Not altered	*χ* ^2^ = 34.12, p = 0.000
	28 (66.7%)	8 (19.0%)	6 (14.3%)	18 (42.9%)	8 (19.0%)	16 (38.1%)	

Results are expressed as number of participants and percentage of the total number of participants.

Positive results were also detected in area of social skills (*χ*^2^ = 17.52, p < 0.01). According to the SSS results, 31 participants exhibited disturbed social interaction skills (six significantly altered) at the baseline. This group was reduced to 14 participants after the intervention (none significantly altered). Similarly, the FrSBe scale detected a decrease in frontal damage disturbance (*χ*^2^ = 34.12, p < 0.01). Ten participants from those classified as pathological at the baseline (n = 28) graduated from this classification after the program.

Participant self-reports exhibited strong program acceptance (Table 3). According to the IMI, participants reported high levels of interest and enjoyment (5.74 ± 0.69), found themselves competent (5.53 ± 0.63) but not pressured (2.07 ± 0.97), and considered the intervention useful (6.31 ± 0.50). In addition, as evidenced by the SUS results, the participants deemed the system highly accessible (80.43 ± 8.01).

**Table 3:** **Usability and motivation reports**

**Scale**	**Values**
*IMI*	
Interest/enjoyment	5.74 ± 0.69
Perceived competence	5.53 ± 0.63
Pressure/tension	2.07 ± 0.97
Value/usefulness	6.31 ± 0.50
*SUS*	80.43 ± 8.01

Results are defined in terms of mean and standard deviation.

## Discussion

This study examined the effectiveness and acceptance of a videogame-based group therapy program for improving SA and social skills among TBI survivors. Overall, results show that the experimental intervention promoted the acquisition of SA, and mainly in perceptions of deficits and, to a lesser extent, in the setting of realistic goals. In addition, concomitant improvements were detected in the development of adequate social and behavioral management skills, which is of particular relevance, as problems in these areas often arise after the occurrence of TBI. Interestingly, the experimental intervention was also deemed motivating and usable.

Improvements in intellectual SA after the intervention were detected by the SADI and corroborated by the PCRS. An integrative approach involving different strategies could have led participants to a better understanding of residual impairments and their implications. Previous research has shown that educational approaches can increase SA, as measured by the SADI [36] and PCRS [37]. Feedback interventions have also been used to increase intellectual SA with promising results, also detected by the SADI [38,39] and PCRS [26,39]. It is important to highlight that clinical changes were detected not only by clinicians via SADI scores but also by relatives and caregivers, as reported by the PCRS. However, classifications of participants provided through both scales were not strictly consistent (Table 2). Differences between questionnaires and varying evaluator characteristics and criteria may have caused these differences. Although clinicians are afforded few interactions with patients who are bound to clinical settings, relatives and caregivers typically spend more time with the patients and can observe reactions at home that differ from those elicited in clinical environments. Interestingly, the PCRS results seemed to match the classification shown by the ‘realistic plan-making’ component of the SADI, which indeed proved to be the most challenging component.

Our results also indicate that the experimental intervention had a significant impact on social skills and behaviors. Post-intervention assessment results show that relatives and caregivers perceived improved social skills and reduced behavioral problems among the participants after the intervention. These results suggest that such interventions may allow participants to learn coping strategies, which may in turn enhance their ability to control emotional and behavioral challenges in the home setting. This is particularly relevant, as individuals with TBI often present deficits in social cognition [13], and the majority of previous research has shown only limited improvements in this area within severe chronic brain injury population [40]. It has been suggested that interpersonal relationships are based not only on social skills but also on self-control and self-regulation. Deficits in SA can cause not only lacking awareness of one’s own impairments but also impaired monitoring of one’s own behaviors and their impacts on others [41]. Previous studies have shown that individuals who have experienced behavioral disturbances following a TBI can exhibit significantly less SA relative to those without behavioral disturbances and that the development of SA is associated with successful psychosocial outcomes [41,42].

The benefits of group therapy after a TBI have been reported in previous studies. Group interventions can offer TBI survivors feedback and support from other individuals with TBI while allowing for the normalization of everyday functioning and social behaviors through engagement with others experiencing similar challenges [43-45]. Various group programs for SA development have been evaluated in the areas of interpersonal and communication skills feedback [46,47], cognitive and behavioral rehabilitation and social skills training [21], and discussion [22]. Interestingly, game-based formats have also been used to provide feedback and cognitive rehabilitation [19,20]. Our results are consistent with these preliminary studies, which reinforce the effectiveness of game-based programs.

Participants reported high levels of engagement and motivation when participating in the program, which may increase adherence to treatments. This is particularly critical among individuals with impaired SA since, as noted before, those who are unaware of their limitations can face difficulties in understanding their need for participation in rehabilitation programs. It is interesting to note that none of the participants voluntarily dropped out from the study.

The limitations of the study must be carefully considered when analyzing the results. First, the sample size (42 participants) can be considered small, though it is larger than those typically involved in state-of-the-art interventions [17]. Second, the demographic and clinical attributes of the sample are inherently linked to specialized neurorehabilitation services offered in the study area, which may restrict the generalization of results. Finally, the SSS is a self-administered questionnaire and its interpretation should be done taken into account the SA deficits of the sample. Since an increase of SA is thought to lead to a more accurate perception of deficits [48], which can lead to depression in some cases [49], a decrease could be expected in the participants’ self-reports of social skills. However, scores in the SSS increased after the therapy. The improvement in SA, as reported by the SADI and the PCRS, together with the group therapy could have led participants not only to increase their social skills but also to perceive this improvement.

The complex construct of SA must be addressed using a multidimensional approach to mitigate cognitive, psychological, social, and behavioral challenges faced by TBI survivors. Various strategies have been used to promote awareness, including neuropsychological programs, psychotherapy, compensatory, and facilitation approaches, structured experiences, direct feedback, videotaped feedback, confrontational techniques, cognitive therapy, group therapy, game formats and behavioral intervention [50]. Experimental integrative approaches involving videogame-based group therapy have been shown to improve SA and to ameliorate social skills and behavior deficits, and such programs are also considered motivating and accessible among users.

## Conclusions

The results of this study suggest that integrative videogame-based group interventions that employ various strategies can improve SA, social skills, and behaviors among TBI survivors in an accessible and motivating manner.

## Abbreviations

TBI: Traumatic brain injury
SADI: Self-Awareness Deficits Interview
PCRS: Patient Competency Rating Scale
SSS: Social Skills Scale
FrSBe: Frontal Systems Behavior Scale
SUS: System Usability Scale
IMI: Intrinsic Motivation Inventory
SA: Self-awareness
